# Sleep Disorders in Children With Autism Spectrum Disorder: Insights From Animal Models, Especially Non-human Primate Model

**DOI:** 10.3389/fnbeh.2021.673372

**Published:** 2021-05-20

**Authors:** Shufei Feng, Haoyu Huang, Na Wang, Yuanyuan Wei, Yun Liu, Dongdong Qin

**Affiliations:** ^1^Department of Pediatric Rehabilitation Medicine, Kunming Children’s Hospital, Kunming, China; ^2^State Key Laboratory of Primate Biomedical Research, Institute of Primate Translational Medicine, Kunming University of Science and Technology, Kunming, China; ^3^School of Basic Medical Sciences, Yunnan University of Chinese Medicine, Kunming, China

**Keywords:** autism, sleep, non-human primate, brain development, animal model

## Abstract

Autism Spectrum Disorder (ASD) is a heterogeneous neurodevelopmental disorder with deficient social skills, communication deficits and repetitive behaviors. The prevalence of ASD has increased among children in recent years. Children with ASD experience more sleep problems, and sleep appears to be essential for the survival and integrity of most living organisms, especially for typical synaptic development and brain plasticity. Many methods have been used to assess sleep problems over past decades such as sleep diaries and parent-reported questionnaires, electroencephalography, actigraphy and videosomnography. A substantial number of rodent and non-human primate models of ASD have been generated. Many of these animal models exhibited sleep disorders at an early age. The aim of this review is to examine and discuss sleep disorders in children with ASD. Toward this aim, we evaluated the prevalence, clinical characteristics, phenotypic analyses, and pathophysiological brain mechanisms of ASD. We highlight the current state of animal models for ASD and explore their implications and prospects for investigating sleep disorders associated with ASD.

## Introduction

Sleep appears to be essential for most living organisms’ survival and integrity, especially for typical synaptic development and brain plasticity (Fogel et al., 2012; Hartsock and Spencer, 2020). Over the past decades, the role of sleep in learning and memory has been probed by many studies at behavioral, systemic, cellular, and molecular levels (Walker and Stickgold, 2006; Rawashdeh et al., 2007; Sawangjit et al., 2018; Kim et al., 2019). The American Academy of Sleep Medicine (AASM) has recently released the third edition of the International Classification of Sleep Disorders (ICSD-3) in 2014. This guideline grouped sleep disorders into seven basic types: insomnia disorders, central disorders of hypersomnolence, circadian rhythm sleep-wake disorders, sleep-disordered breathing, movement disorders, parasomnias, and other sleep disorders (Sateia, 2014; Ito and Inoue, 2015). There are universal physiologic changes during sleep, and some biologic, environmental, psychological, social as well as genetic factors can affect change in the sleep pattern (Jenni and O’Connor, 2005; Bathory and Tomopoulos, 2017). The sleep-wake circadian rhythm is regulated through both circadian and homeostatic processes. Arousal and sleep are active and involved in neurophysiologic processes, including both activation and suppression of neural pathways. Sleep disorders can be an early symptom of the disease, and the presence of rapid eye movement (REM) sleep behavior disorder (RBD) can be used as an early diagnostic indicator for neurodegenerative diseases (Galland et al., 2012; Kotterba, 2015). Besides, sleep disorders have observable effects on physical and mental health of children with autism spectrum disorder (ASD) and their parents (Zhang et al., 2021).

Autism spectrum disorder is a neurodevelopmental disorder and the prevalence of ASD is increasing, with 1 in 59 children in the United States. diagnosed with ASD (Orefice, 2019). ASD is approximately four times more prevalent among males than females (Christensen et al., 2016; Satterstrom et al., 2020). According to the DSM-V, the previous categories of pervasive developmental disorders, pervasive developmental disorder-not otherwise specified (PDD-NOS) and Asperger disorder were combined into ASD. The new diagnostic criteria from DSM-V defined ASD as a heterogeneous spectrum disorder with deficits in social interaction and communication, restricted and repetitive interests, and stereotyped behaviors (American Psychiatric Association, 2013; Devnani and Hegde, 2015).

It is not surprising when considering the numerous health and behavioral issues that sleep disturbance are commonly observed in the clinical progression of ASD. Children with ASD experience more sleep problems compared with the general population, particularly insomnia. Sleep-wake cycle abnormalities are associated with communication deficits, stereotyped behaviors, and autism severity (Tudor et al., 2012). Disrupted sleep may exacerbate the daily dysfunction of ASD children, such as social and communication skills, behavioral performance, stereotypical behaviors, and motor output on non-verbal performance tasks (Schreck et al., 2004; Limoges et al., 2013).

As we gain deeper knowledge of the neural mechanisms of ASD and sleep, more contributions from sleep-related biomarkers to the study of neurophysiology in ASD. Prospectively, the emergence of digital technologies and devices is making studies of sleep physiology more flexible and convenient. The sleep study provided new insights for research on the children with ASD when compared with the other behavioral tests currently used in human subjects and animal experimental models. This review’s main objective is to explore animal models’ role, especially non-human primate (NHP) models, as a useful tool to investigate sleep disorders in ASD children. Firstly, we present data on sleep disorders in autistic children, emphasizing their prevalence, clinical characteristics, phenotypic analyses, and pathophysiological mechanisms. Next, we highlight the current state of animal models for ASD and explore their implications and future prospects in translational research. We suggest that using NHP animal models may provide insights into sleep disorders in ASD.

## The Role of Sleep

In most mammalian species, sleep amounts are highest during the neonatal period (Weber and Dan, 2016). Sleep loss can significantly affect a child’s health-related quality and activities of daily living (Maski and Kothare, 2013). The brain is one of the organs most impacted by sleep or the lack thereof while adolescence is a critical period for brain reorganization. It is beyond doubt that sleep disorders during this period exert irreversible effects on children’s brain development (Roffwarg et al., 1966; Klein et al., 2000; Weber et al., 2018). REM sleep can prune newly formed postsynaptic dendritic spines during motor learning (Li et al., 2017), and the balance of newly formed and original dendritic spines is crucial for neuronal circuit development and behavioral improvement in children. Two studies found that sleep enhance cortical plasticity in the visual cortex during the developmental critical period (Frank et al., 2001; Aton et al., 2009). In conclusion, sleep seems to be important for brain development, learning, and memory consolidation by selectively eliminating and maintaining newly formed synapses (Li et al., 2017).

Sleep deprivation may cause physical diseases and developmental problems. During early life, sleep deprivation has been shown to have long-term implications for social behaviors in adulthood (Hudson et al., 2020). Neural substrates can be affected by sleep deprivation, including the prefrontal cortex, basal ganglia, and amygdala. Furthermore, sleep deprivation may cause difficulties in executive functioning, reward learning as well as emotional reactivity. Such issues may contribute to difficulties in judgment, resolution of problems, challenging behaviors, emotional control, and public health concerns, such as depression, suicide, and risk-taking behavior (Maski and Kothare, 2013). These findings indicate that insufficient sleep during early life has persistent effects on brain development and later behavioral performance.

It has been assumed that sleep can clear out brain’s toxins, such as beta-amyloid which was associated with Alzheimer’s disease (Xie et al., 2013). Sleep is essential for maintaining the body’s physical health and is associated with neurodegeneration, metabolic diseases, cancer, and aging. The processes of growth and development are related to sleep quality. The abnormal sleep and circadian also affect hormones and metabolism (Li et al., 2018a). Getting adequate sleep can help the immune system to better react against infection (Grigg-Damberger, 2009; Herculano-Houzel, 2013; Welberg, 2013).

## Clinical Characteristics of Sleep Disorders in ASD Children

Many neurodevelopmental processes have been reported in the children with ASD, such as synaptic plasticity, neurogenesis and migration of neuron (Gilbert and Man, 2017). About 40–80% of children with ASD exhibit at least one sleep-related problems (Verhoeff et al., 2018), including irregular sleeping and waking patterns, decreased sleep efficiency, reductions in total sleep time and REM sleep time, sleep onset delays, decreased sleep efficiency, increased wakening after sleep onset, bedtime resistance, and daytime sleepiness (Humphreys et al., 2014). Studies utilizing Actigraphy (ACT) and Polysomnogram (PSG) have found that increased sleep latency, and decreased sleep duration and sleep efficiency in ASD children (Elrod and Hood, 2015). A comprehensive review in children with ASD reported that insomnia is one of the most common sleep problems (Souders et al., 2009). Another study also documented that the predominant sleep disorder included insomnia, difficulty falling, and staying asleep (Malow et al., 2006). Mutluer et al. (2016) found that the most common symptoms reported were troubles falling asleep, sleep after waking up and tired after sleeping.

## Prevalence of Sleep Problems in Children With ASD

Childhood sleep disorders which are mostly reported by parents are associated with emotional, cognitive, and behavioral disturbances. Sleep disturbances occur in approximately 20–30% of preschool children, including bedtime resistance, sleep onset delays, night terrors or nightmares, and repetitive rhythmic behaviors (Lozoff et al., 1985; Krakowiak et al., 2008; Knappe et al., 2020). The abnormalities of ASD may predispose children to various threaten of sleep and make them especially susceptible to sleep problems (Morgenthaler et al., 2007; Maxwell-Horn and Malow, 2017). Sleep problems have become one of the most common symptoms among ASD children (Richdale, 1999; Wiggs, 2001; Liu et al., 2006; Uren et al., 2019). Two studies compared sleep behaviors of ASD with typically developing (TD) children, they found that 66% of ASD children exhibited moderate sleep disturbances (Souders et al., 2009) and 71% in another study (Malow et al., 2016). A parent-reported study found that 35% of ASD children had at least one sleep dysfunction (Krakowiak et al., 2008). The risk of sleep disturbance is 2.8-fold higher in children with ASD (Köse et al., 2017). A recent study repeated sleep measures at different age in 5,151 children, and found that ASD children have an increase in sleep problems with age, whereas TD children decrease (Verhoeff et al., 2018).

## Phenotype Analyses for Sleep Disorders

**FIGURE 1:**
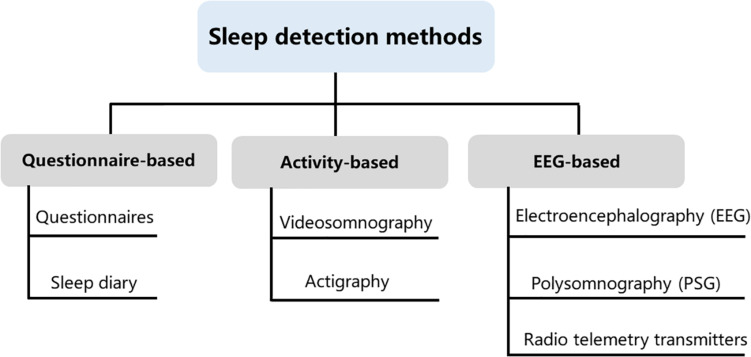
Sleep detection methods. Current methods available for measuring sleep in young children include questionnaire-based, activity-based, and electroencephalogram (EEG)-based methods. These three types of assessments are not interchangeable, as each method contains its own idiosyncrasies that can influence the quality and meaning of the data that are collected.

Over the past years, many different sleep analysis methods have been reported (Lomeli et al., 2008; Kelly et al., 2012; Ibáñez et al., 2018; de Zambotti et al., 2019). Infection, pain as well as trauma can disrupt sleep and activity (Vandekerckhove and Cluydts, 2010; Doufas et al., 2012), even some issues that might seem minor to us are often very significant to a child. As children progress from infancy to adolescence, sleep structure, sleep behavior and sleep duration will also change (Williams et al., 2013), it is crucial to take into account the specificity of different ages of children when investigating sleep states. Some sleep studies require an intimate contact of the electrode with the skin and even require surgical implantation of electrodes, which are difficult to apply in freely moving animals and humans, particularly in children related to the lively side of their nature. Even though several new technological developments have been brought to reduce inconvenience, pain, and further damage of these methods, expensive and burdensome must also be considered, especially for long-term studies that include large samples. Some assessments were developed to monitor sleep through observation of body motion and posture. These methods could obviate the need for direct contact and even avoid surgery or electrode implantation. It is non-invasive and low cost. Nevertheless, the behavioral observation does not provide sufficient information compared to those provided by electroencephalogram (EEG) and electromyogram (EMG). In general, both humans’ and animals’ sleep analyses include sleep patterns, locomotor activity, temperature, and food intake. The current study summarized phenotype analyses for sleep disorders obtained from sleep diaries, parent-reported questionnaires, electroencephalography, actigraphy, and videosomnography. We summarized the main types of experimental approaches applicable to assessment methods of sleep studies and all of these methods have advantages and disadvantages (Figure 1). The selection of clinical sleep assessment should be tailored to children’s unique characteristics, and safety and feasibility must also be taken into consideration.

### Sleep Diaries and Parent-Reported Questionnaires

Subjective measures including sleep diaries and parent-reported questionnaires are the most common analyses in human studies. They have several advantages such as the non-invasive ease of acquisition and low cost. Parents are usually quick to recognize any changes in their child’s behavior and mood, and these observations should be recorded (Bhargava, 2011). One of the most common parent-reported questionnaires is Children’s Sleep Habits Questionnaire (CSHQ), a parent-report sleep screening instrument designed for school-aged children. The CSHQ score has eight measures, evaluating the behavioral and psychological symptoms of sleep disorders in children (Owens et al., 2000). Many factors affect the reliability of sleep analysis. These factors include the aspect of sleep assessed, the period of sleep aggregated, and the sample population and so forth (Acebo et al., 1999; Camerota et al., 2018). Most retrospective studies on sleep are reported by proxies, such data are limited by problems related to recall bias and subject selection bias (Short et al., 2017). When parents have to estimate the sleep habits of their children, it has been shown that parents tended to estimate with more accurate sleep schedule variables than time awake in bed (Werner et al., 2008). Moreover, the consistency of their reports decreased when the monitoring lasted a long time (Short et al., 2013). Although biases are introduced when utilizing these methods, sleep diaries, and parent reports are commonly used in monitoring children with sleep problems because of their low cost and ease of administration (Honomichl et al., 2002).

### Electroencephalography

The influence of technology advances becomes increasingly evident in the study of neuroscience. EEG can provide the temporal and spatial characteristics of subject. A sleep EEG is a recording of the electrical activity of the brain while you are awake and then asleep. It involves having small disks (electrodes) which record the activity attached to the subject’s scalp (Feinberg et al., 1967; Weber and Dan, 2016). Compared with a single-channel EEG, one technique named polysomnogram (PSG) is considered as the gold standard to objectively assess sleep (O’Donnell et al., 2018). PSG can be used in a diverse range of monitoring, such as brain electrical activity, muscle activity, eye movements, respiratory rate and other channels relying on experimental design (Boulos et al., 2019). It integrates both normal and abnormal physiological indicators of brain electrical activity, sleeps architecture, sleep stages, quality of sleep, eye movements, and physical activities during the sleep period. Wakefulness, NREM sleep, and REM sleep can be clearly distinguished making sleep a directly quantifiable behavior, which could be introduced more easily into clinical routine and less stressful for patients (Blume et al., 2015). The main drawback of PSG is the need of electrodes attached to the skin surface, and not convenient to use in clinical sleep monitoring for children (Lucey et al., 2016). The children cannot be sedated by given medicine such as tranquilizers or sleep aids during the PSG sleep study, and thus doctors may use a blanket or papoose board to keep the child from rolling around on the bed or pulling on the wires. However, this issue may restrict children’s normal sleep as we don’t expect. And also, PSG instruments are bulky and expensive and may be difficult to monitor changes in patients for long-term studies (Stepnowsky et al., 2013; Qin et al., 2020). Recently, telemetry transmitters have been used for long-term measuring of EEG and electromyography signals in rodent and NHP animals, it could collect data from conscious, freely moving laboratory animals without skin-electrode contact impedance and reduce animals’ stress (Ishikawa et al., 2017; Qiu et al., 2019). This strategy can be potentially applied for future clinical applications.

### Actigraphy

Actigraphy is a non-invasive method that measures limb movement by a watch-size accelerometer to determine sleep and wake episodes. It allows for multiple-day data collection in natural environments. One study compared the validity of actigraphy and PSG, found that intraclass correlations between PSG and actigraphy variables were strong (>0.80) for sleep latency, sleep duration, and sleep efficiency (Bélanger et al., 2013). Nevertheless, lack of correspondence of circadian sleep-wake cycles between actigraphy and PSG was confirmed in school-age children (Meltzer et al., 2016). Actigraphy assessments may severely underestimate the true sleep statements in children with significantly elevated sleep disorders (Sadeh, 2011).

The next generation multisensory consumer sleep trackers are different from the first motion-based generation of consumer wearables (actigraphy). New generation sleep trackers apply algorithms to achieve functions approximately similar to PSG. Fitbit (Montgomery-Downs et al., 2012; Meltzer et al., 2015; de Zambotti et al., 2016; Mantua et al., 2016; Cook et al., 2017; de Zambotti et al., 2018) and Jawbone (de Zambotti et al., 2015; Toon et al., 2016; Cook et al., 2018) sleep trackers are most frequently tested wearables and their performance has always been compared with PSG. Boe et al. (2019) recently presented a wireless, wearable sensor measuring hand acceleration, electrocardiography (ECG), and skin temperature that outperforms the ActiWatch (one common equipment of actigraphy), detecting wake and sleep with a recall of 74.4 and 90.0%, respectively.

### Videosomnography

For centuries, many videosomnography monitoring systems have been used to measure predefined daily activities continuously (von Ziegler et al., 2021). Like actigraphy, the advantages of videosomnography lie in its objective documentation for long-term interval (Goodlin-Jones et al., 2001; Burnham et al., 2002). It can also be used for capturing abnormal events such as parasomnias during night. However, there are several challenges using videosomnography in sleep research for children. First of all, the portable systems that capture time-lapse video recording are expensive and often need laborious and subjective human labeling. Additionally, camera is placed in fixed positions, the angle of review and the motion of children may affect the quality of video recording. Finally, ethical concerns and privacy issues of videosomnography surveillance system must be considered (Sadeh, 2015; Schwichtenberg et al., 2018). Videosomnography is now widely used in animal sleep research. Most non-invasive rodent sleep assessments depend on gross body movement (Pack et al., 2007; Fisher et al., 2012). Three-state sleep staging can be recorded by using electric field sensors to capture both gross body movement and respiration-related measures (McShane et al., 2012; Mingrone et al., 2020). For NHP study, sleep states are judged by focusing on two major behavioral features: whether the eyes were open or closed, and whether gross movements were present or absent (Prechtl, 1974; Mizuno et al., 2006; Chen et al., 2017). Over the past years, software packages based on deep learning/neural networks allow marker less tracking of multiple, hand-picked body points with astonishing performance.

## Animal Models Used in the Study of ASD

Numerous animal models of ASD have been generated in the last decade (Peñagarikano et al., 2011; Li et al., 2015; Kazdoba et al., 2016; Sacai et al., 2020). Many ASD-associated genes such as Neuroligins play a crucial role in regulation of synaptic adhesion and keeping imbalance between excitatory and inhibitory control in brain circuits (Wintler et al., 2020). The gene editing tools have rapidly been adopted by scientists to parse the role of genetic abnormalities in the etiology and symptomology of ASD. Because the more established gene editing technologies were used in the mice, mice have become the primary animal model of genetic diseases (Crawley, 2012). Growing studies of NHP models have been generated because their close phylogenetic relatedness to humans (Gadad et al., 2013). Moreover, mounting evidence suggests that environmental factors during early development is important. Animal models of maternal exposure to valproic acid and maternal immune activation appear to be the most commonly used. Frequent blood draws and PSG recordings, which are difficult procedures for children with ASD, also make the ASD models becoming ideal candidates. Here, we summarize some rodent (Table 1) and NHP (Table 2) models of ASD, which may have potential value to investigate the causes and effects of ASD, as well as their effects on brain development and sleep disorders.

**TABLE 1:** Autism-relevant phenotypes in selected rodent models.

**Models**	**Methods**	**Ages**	**Phenotypes**	**Sleep disorders**	**Brain development**	**References**
Genetic rodent models	Cntnap2 knockout	7 days to 6 months	Abnormal social contact, hyperactivity and epileptic seizures. Increased repetitive behaviors and reduced juvenile ultrasonic vocalizations	Wake fragmentation and reduced spectral power in the alpha (9–12 Hz) range during wake	Impaired neuron migration and abnormal neural network connectivity	Peñagarikano et al., 2011; Thomas et al., 2017
Genetic rodent models	Neuroligin-1 (NLG1) knockout	2–8 months	Impaired social approach, repetitive behavior and deficits in spatial learning	NLG1 knockout mice do not sustain wakefulness and spend more NREM sleep. Low theta/alpha activity during wakefulness and altered delta synchrony during sleep	Abnormal long-term potentiation in hippocamp and decreased ratio of NMDA/AMPA glutamate receptor at cortico-striatal synapses	Blundell et al., 2010; El Helou et al., 2013
Genetic rodent models	Neuroligin-2 (NLG2) knockout	5–8 weeks	Increased anxiety-like behavior, decreased pain sensitivity, motor coordination, exploratory activity and ultrasonic pup vocalizations. Developmental milestone delays	More wakefulness and less NREM and REM sleep. Abnormal “hyper synchronized” EEG events during wakefulness and REM sleep	Reduced inhibitory synaptic puncta and impaired synaptic neurotransmission	Blundell et al., 2009; Wöhr et al., 2013; Seok et al., 2018
Genetic rodent models	Neuroligin-3 (NLG3) knockout	50–70 days	Reduced fear conditioning. Olfactory impairments and hyperactivity. Reduced ultrasound vocalization and social novelty preference	Significantly impaired EEG power spectral profiles during wake and sleep	Increased inhibitory neuro-transmission in the barrel cortex, enhanced long-term potentiation in the hippocampus. Decrease of total brain volume	Radyushkin et al., 2009; Liu et al., 2017
Genetic rodent models	Shank3 knockout	4–88 days	Repetitive grooming, Abnormal social interactions and vocalizations, and reduced open field activity	Reduced sleep intensity and delayed sleep onset	Impaired long-term potentiation. Impaired transmission and plasticity in hippocampus. Deficits in baseline NMDA receptor-mediated synaptic responses	Jaramillo et al., 2016; Dhamne et al., 2017
Environmentally-induced models	exposure to valproic acid (VPA) during pregnancy	7–40 days	Social behavioral deficits, increased repetitive behavior, and impaired communication	More wake and NREM sleep, disrupt sleep architecture. Decreased theta and increased gamma power during REM sleep	Decreased cortical levels of GAD65 and GAD67—markers of GABAergic synapses. Increased basal levels of serotonin	Tsujino et al., 2007; Nicolini and Fahnestock, 2018
Environmentally-induced models	Pregnant mice infected with virus or synthetic dsRNA, poly(I:C)	7–12 weeks	Reduced social behavior and increased anxiety-like behavior	Abnormal EEG power and spontaneous epileptiform activity	Deficits in synaptic strength of prefrontal to amygdala neural circuits. Increases in microglia and neuro-inflammatory markers	Li et al., 2018b; Missig et al., 2018

**TABLE 2:** Autism-relevant phenotypes in selected primate models.

**Models**	**Methods**	**Ages**	**Phenotypes**	**Sleep disorder**	**Brain development**	**References**
Rett Syndrome	MECP2 mutations mediated by TALENs	7–8 months	Increased sensory threshold and stereotypical behaviors, social communication deficits and abnormal eye-tracking	Sleep in mutants was more fragmented. Significantly longer awake durations and shorter total sleep durations	Significantly reduced cortical gray matter and white matter. Reduced total cortical volumes and thicknesses	Chen et al., 2017
MECP2 duplication syndrome	MECP2 overexpression by lentivirus-based transgenic	12–18 months and then to 55 months	Increased repetitive behavior and stress responses. Reduced social contact	N/A	Reduced β-synchronization within frontal-parieto-occipital networks. Hypoconnectivity in prefrontal and cingulate networks	Liu et al., 2016; Cai et al., 2020
Maternal immune activation	Poly IC injection	6–24 months	Increased repetitive behaviors, communication deficits, abnormal social interactions and affiliative calls	N/A	Altered dendritic morphology. Reduces in both gray matter and white matter. Alterations of dendritic morphology	Bauman et al., 2014; Machado et al., 2015
Maternal immune activation	Valproic acid (VPA) explored	17–21 months	Abnormal social interaction, increased stereotypies, and abnormal eye-tracking	N/A	Severe neurogenesis defects and abnormal neurogenesis	Zhao H. et al., 2019
SHANK3 mutation	CRISPR/Cas9	1–12 months	Motor deficits and increased repetitive behaviors. Social and learning impairments	Increased sleep latency and nocturnal waking. Reduced sleep efficiency	Decreased gray matter. Dysregulated resting-state brain connectivity	Zhou et al., 2019

### Rodent Models for ASD

The CNTNAP2 gene encodes cortactin-associated protein-like 2 (CASPR2), which is a cell adhesion molecule and receptor (Jackman et al., 2009). Research of CNTNAP2 demonstrated a connection between genetic risk for autism and specific brain structures (Alarcón et al., 2008). A linkage study reported an increased familial risk for autism with mutations of the CNTNAP2 gene (Arking et al., 2008). Cntnap2 knockout (KO) mice have very similar presentations as with ASD including hyperactivity and epileptic seizures. Analyses of these mice indicated abnormal neuronal migration and synchrony (Peñagarikano et al., 2011).

Neuroligins (NLs) are a diverse class of proteins distributed molecules with functions of excitatory or inhibitory synapse specification (Ichtchenko et al., 1995; Ichtchenko et al., 1996; Graf et al., 2004; Blundell et al., 2010). Neuroligin-1 (NLG-1) is enriched preferentially at excitatory synapses (Song et al., 1999), neuroligin-2 (NLG-2) is enriched at inhibitory synapses (Varoqueaux et al., 2004; Levinson and El-Husseini, 2005), and neuroligin-3 (NLG-3) appears to be present at both (Fekete et al., 2015). The activity of NLG1 is impaired by prolonged wakefulness. Neuroligin-1 is related to neuronal activity and associated with regulation of sleep and wake (El Helou et al., 2013). Janine et al. found that NLG-1 knockout mice can hardly sustain wakefulness and spend more time in NREM sleep. Neuroligin-2 knock-out mice have less total sleep time and exhibit abnormal spike and wave discharges and behavioral arrests characteristic of absence seizures (Cao et al., 2020). Neuroligin-3 knock-out mice exhibit reduced fear conditioning, olfactory impairments and hyperactivity, as well as reduced ultrasound vocalization and social novelty preference (Radyushkin et al., 2009; Liu et al., 2017).

It has been proven that SHANK3 may induce sleep difficulties in patients with ASD. SHANK proteins are important organizers for signaling proteins in the post-synapse of excitatory neurons. In neurons, SHANK2 and SHANK3 have a positive effect on the induction and maturation of dendritic spines, whereas SHANK1 induces the enlargement of spine heads. Patients with an ASD-associated condition called Phelan-McDermid syndrome (PMS) are often missing the SHANK3 gene and they also often have sleep problems (Phelan and McDermid, 2012; Bro et al., 2017; De Rubeis et al., 2018). A recent meta-analysis of SHANK mutations suggested that SHANK3 mutations have a higher frequency and penetrance in individuals with ASD, compared to SHANK1 and SHANK2 (Leblond et al., 2014). Shank3 mutant mice show a variety of features of both ASD and PMS (Jaramillo et al., 2017; Ingiosi et al., 2019). In Shank3 heterozygous mice, there was a reduction in basal neurotransmission (Bozdagi et al., 2010). Shank3 knockout mice exhibit many autistic-like behaviors such as repetitive grooming, social deficits, reduced activity, anxiety-related behavior, as well as learning and memory impairments (Jaramillo et al., 2016; Dhamne et al., 2017). Shank3 KO mice have reduced sleep intensity and delayed sleep onset.

Overall, there were many other rodent models of ASD displaying reduced sleep time: 16p11.2, Fmr1, Mecp2, Ube3a, Rims1, Scn1a, Scn8a, Disc1, Gabrb3,Camk2a, Cacna1g, and Npas2 (Dudley et al., 2003; Lee et al., 2004; Anderson et al., 2005; Lonart et al., 2008; Kimura et al., 2010; Papale et al., 2010; Johnston et al., 2014; Zhou et al., 2014; Ehlen et al., 2015; Kalume et al., 2015; Kumar et al., 2015; Jaramillo et al., 2016; Tatsuki et al., 2016; Dittrich et al., 2017; Lu et al., 2019). Although the majority of these mutant rodent models exhibit reduced activity, which could be indicative of decrease sleep duration, the prevalence of serious sleep problems such as sleep fragmentation is far less than what has been observed in the clinical population.

While there is strong genetic effect, the etiology of ASD seems to be multifactorial. Environmental factors including toxins, pesticides, infection, and drugs also have a strong correlation. Environmental exposure during preconception, prenatal, and postnatal pregnancy can impact the immune system and the developing nervous system, and may cause neurodevelopmental disorders including ASD.

Valproic acid (VPA) is a drug used in humans primarily for epilepsy and seizure control. VPA is currently considered to be a risk factor for ASD and is also known teratogenicity (Balfour and Bryson, 1994). It has been demonstrated that exposure to VPA during pregnancy would increase the risk of autism in children based on several studies in humans (Laegreid et al., 1993; Christianson et al., 1994) and experimental evidence in animals (Lin et al., 2013). Furthermore, rodents prenatally exposed to this drug exhibit autism-like behavior including social behavioral deficits, repetitive and stereotypic behaviors, and impaired communication (Mychasiuk et al., 2012; Nicolini and Fahnestock, 2018). Intraperitoneal injection of VPA to rats with pregnancy would make their offspring exhibiting autism relevant behavioral and physiological indicators (Schneider et al., 2008).

Several studies have reported correlation between maternal antibody reactivity toward fetal brain proteins and ASD in the children (Braunschweig et al., 2008; Croen et al., 2008; Brimberg et al., 2013). In the rodent maternal immune activation model of ASD (Smith et al., 2007; Malkova et al., 2012; Choi et al., 2016; Kim et al., 2017), offspring from pregnant mice which were infected with virus or injected intra-peritoneally with synthetic dsRNA [poly(I: C)], exhibited behavioral symptoms such as social deficits, communication deficits, and repetitive behaviors. For brain neuropathology, the offspring of maternally infected mice displayed significantly fewer Purkinje cells. These data are quite similar to both ASD behavioral and neuropathological phenotypes.

### Non-human Primate Models

Non-human primates are among the optimal animal models, in large part because of their close phylogenetic relatedness with humans (Zhang et al., 2014; Nunn and Samson, 2018). With the rapid advances in gene-editing technologies, researchers have established several NHP models for ASD (Liu et al., 2016; Sato et al., 2016; Chen et al., 2017; Tu et al., 2019). It would be valuable for researchers to be attentive to study of many kinds of disease by using NHP animal models (Anderson, 2000).

MECP2 duplication syndrome is an X-linked recessive syndrome resulting from abnormal genomic rearrangement. The two major clinical symptoms are intellectual disability and anxiety. MECP2 overexpressed monkey models exhibited characteristic features of ASD such as social deficits, repetitive behaviors, and increased anxiety (Liu et al., 2016). Cai et al. reported a combination of gene-circuit-behavior analyses, including MECP2 co-expression network, locomotive and cognitive behaviors, and EEG and fMRI findings in MECP2 overexpressed monkeys. Whole-genome expression analysis revealed MECP2 co-expressed genes were significantly enriched in GABA-related signaling pathways, whereby reduced β-synchronization within frontal-parietal-occipital networks was associated with abnormal locomotive behaviors (Cai et al., 2020).

Rett syndrome caused by mutations in MECP2 is a prototypical neurodevelopmental disorder. Researchers demonstrated that MECP2 mutant monkeys could well mimic autism-associated abnormalities in physiology and social behavior (Chen et al., 2017). The mutant monkeys exhibited significantly increased total awake time and more fragmental sleep during night, which have also been found in Mecp2 mutant mice (Li et al., 2015).

Feng et al. used CRISPR/Cas9 to generate SHANK3, a top autism gene mutant monkey. SHANK3 mutant monkeys tend to be less active and have troubles sleeping that they take longer time to fall asleep and wake up more often. Monkeys in this study have severe repetitive movement, deficient social skills, and show brain-activity patterns similar to those seen in autistic people (Le Bras, 2019; Tu et al., 2019). SHANK3-deficient monkeys showed reduced spine density and impaired development of mature neurons in the prefrontal cortex (Zhao et al., 2017). It has also been found that some rhesus macaques carried spontaneous mutation of SHANK3 (Vegué and Roxin, 2015). Spontaneous mutations in NHPs may have the potential to be used as a suitable animal model to figure out the relationships between genetic variants and behaviors (Haus et al., 2014; Zhao et al., 2018).

Rodent animal models of maternal exposure to VPA provided evidence that environmental risk factors in ASD. Recently, Zhao H. et al., 2019 reported the neurodevelopmental and behavioral outcomes of maternal VPA exposure in NHP for the first time. Offspring from maternal exposure to VPA has significantly impaired neuronal development. VPA-exposed monkey offspring showed impaired social interaction, communication disabilities, and abnormal eye-tracking (Zhao H. et al., 2019).

When rhesus monkeys were given the viral mimicking synthetic double-stranded RNA (polyinosinic:polycytidylic acid stabilized with poly-L-lysine) during pregnancy, and their offspring could exhibit abnormal repetitive behaviors, altered communication, impaired social interactions and abnormal gaze patterns to salient social information (Bauman et al., 2014; Machado et al., 2015). These offspring with autism-like behaviors also have reduced gray matter in most of the cortex and decreased white matter in the parietal cortex (Short et al., 2010). Novel evidence implicating MIA exposure with alterations of NHP dendritic morphology have been found (Weir et al., 2015). The mother and the fetus exploit several mechanisms in order to avoid fetal rejection and to maintain an immunotolerant environment during pregnancy. The placenta is an important organ that facilitates nutrient exchange. It has been reported that the anatomy of the placenta is varied across species, and it is highest in humans, intermediate in rhesus macaques, and minimal in rodents (Carter, 2007). Thus, the role of the NHP animal model in this field of research is important.

## Monkeys as an Ideal Animal Model for Studying Sleep in ASD

An ideal animal model of human disease should show tight junctions with clinical characteristics of the disease. The statistics from United States government in 2010 indicated that almost 90% of the laboratory animals used in science research are mice, rats, and other rodents. NHP only represents 0.28% among all animals (Phillips et al., 2014). However, rodents diverged from humans by more than 70 million years of evolution. There are significantly evolutionary differences in brain anatomy, cognitive capacity, and social behavior between humans and rodents (Kumar and Hedges, 1998; Gibbs et al., 2004). Compared with rodents, rhesus macaque (Macaca mulatta), most common NHP used in study, are separated from humans approximately 25 million years ago and are more similar to humans in genetics, neurobiology, and behavior. Thus, NHP have reasonable behavioral correlates to the characteristics of patients in ASD, such as repetitive behaviors, communication deficits, and stereotyped behavior (Watson and Platt, 2012; Parker et al., 2018). As mentioned previously, prenatal environment and gestational timing may impact neurodevelopment of offspring. The gestational period of rhesus monkeys (165 days) and humans (280 days) is much longer than mouse (18–23 days) (Clancy et al., 2001). Besides, the prenatal immune challenge and neuron development of primates occur mostly during the third trimester of prenatal and during early postnatal period (Careaga et al., 2017). The mouse is becoming increasingly popular for genetic studies. However, the mouse’s brain weighs a few grams, and ours weighs one and a half kilos. Can we use the mouse to learn something about our brain? The region of the neocortex is almost 80% in the human brain, which is just 28% in the rat (Roberts and Clarke, 2019). Human prefrontal cortex includes granular and agranular cortex, while rat prefrontal cortex only contains agranular cortex (Ongür and Price, 2000; Uylings et al., 2003). It has been proposed that the prefrontal cortex has a substantial role in social processing, and its potential dysfunction may cause ASD (Sugranyes et al., 2011). The temporal lobe is a morphological brain region which is unique to primates (Colombo et al., 2000; Bryant and Preuss, 2018). The major areas of the human brain classified by Brodmann have also been identified in NHP. Structure and function of the amygdala are nearly the same in the human and non-human primate (Gowen et al., 2007; Rutishauser et al., 2015; Schumann et al., 2016), but remarkably different from the rodent brain (Chareyron et al., 2011). The close relationship of development and evolution between NHP and human show that great prospects to mimic clinical realities by designing NHP animal models.

As mentioned previously, sleep problems in children with ASD are caused by multi-factorial risks such as abnormal neurodevelopment and environmental factors (Bourgeron, 2007; Owens and Mindell, 2011). Modeling clinical disorders in animals provide an opportunity to improve translational research although the human disorder’s clinical phenotype is complex and heterogeneous and lacks objective homologous endpoints across species (Missig et al., 2020). Many previous studies of ASD animal models exhibited several hallmark features which have been documented in humans.

Sleeping studies in humans must be done in accessible samples, predominantly saliva or blood, and confounded by environmental factors. Species-specific differences including light and biological rhythm, as well as sleep features have been noted in studies of sleep (Campbell and Tobler, 1984). For example, rodents are commonly thought to awake during the dark phase and asleep during the light phase. However, researchers found that mice are not explicitly nocturnal, and they have diurnal feeding activity. Researchers also reported that seasonal influences were demonstrated to be more potent on activity than specific genes which was generally considered to control sleep (Daan et al., 2011). Effective use of animals to study normal sleep and sleep disorders must consider known similarities and differences between human and animals. Likewise, sleep is important to keep health and can significantly influence daily activity schedules in NHP (Fruth et al., 2018; Qiu et al., 2019). Sleep structure and EEG patterns of NHP are closely related to the consolidated and monophasic organization observed in humans (Reite et al., 1965; Hsieh et al., 2008), which contrasts with the more fragmented sleep patterns in rodents (Fifel and Cooper, 2014) (Table 3). NHP is also a diurnal animal to better recapitulate clinical conditions with behavioral and metabolic properties closer to humans. Humans pass through 4–6 cycles of NREM and REM within a night’s sleep, which are much shorter in rats and mice (Fuller et al., 2006; Toth and Bhargava, 2013). Nunn and Samson compared sleep patterns in 30 different species of primates, including humans. Most species generally sleep between 9 and 15 h, while humans averaged just 7 h (Nunn and Samson, 2018). In summary, measuring behavioral and sleep states in NHP may provide a better understanding of sleep disorders in children with ASD compared with rodents.

**TABLE 3:** Different sleep pattern between human and animals.

	**Human**	**Monkey**	**Rat**	**Mice**
Primary circadian sleep phase	Dark	Dark	Light	Light
Sleep pattern	Monophasic or diphasic	Monophasic or diphasic	Polyphasic	Polyphasic
Total sleep duration (24 h)	6–8 h	9–12 h	12–15 h	12–15 h
Sleep efficiency (%) (12 h dark)	95%	88%	55%	33%
REM sleep (%) (12 h dark)	20–25%	28%	7–9%	3–5%
NREM sleep (%) (12 h dark)	60–83%	76–80%	26–30%	22–29%
References	Roffwarg et al., 1966; Campbell and Tobler, 1984; Carskadon and Dement, 2005	Hsieh et al., 2008; Authier et al., 2014; Rachalski et al., 2014; Ishikawa et al., 2017; Qin et al., 2020	Zepelin et al., 1972; Seelke and Blumberg, 2008	Vyazovskiy et al., 2006; Hasan et al., 2012

## Current Challenges

Autism spectrum disorder is a neurodevelopmental disorder and the origins of ASD remain unresolved. The potential estimates including genetic, maternal, and environmental effects (Bai et al., 2019). At first, there are various types of genetic variation, such as single-nucleotide polymorphism (SNP) or rare genetic mutations (Weiner et al., 2017). Animal model studies have shown that the impact of genetic on behavior is complex and not completely correspond to specific behavior. Brain development can be influenced by not only the expression of genes, but also modified by environmental factors during the pregnancy and postnatal period. Therefore, the application of gene editing technology in animal models of disease may not completely mimic clinical phenotypes of humans. Secondly, although the NHP animal model has been used to study the impact of environmental modifications on the brain development. Genetic influences may also affect individual responses to different situations and different types of environmental challenges (Machado and Bachevalier, 2003). In this area, rodent models may be more appropriate which have more identical genetic backgrounds compared with NHPs. Besides, NHP exhibit significant and stable individual differences in social commination (Capitanio, 1999; Capitanio and Widaman, 2005).

Non-human primates have much longer reproductive cycle and lower reproduction efficiency compared with rodents, which may bring the difficulties to prepare an adequate quantity of experimental animals. Furthermore, the giant body size of NHP may cause a significant challenge for experimental design. Limitations associated with the gene-editing technique, including editing efficiency, chimeras, and off-target effects, should also be brought to attention (Niu et al., 2014; Chen et al., 2016). Lastly, NHP, rather than other animals, require more significant ethical consideration because its significant cognitive capacity and complex social behavior. Researchers have moral responsibility to ensure that experimental animals receive reduced negative effects and suffering (Bentham, 1996; Beauchamp and Frey, 2011). Animal experiments should follow the principles of the 3Rs, including replacement, reduction, and refinement (Russell and Burch, 1959; Jennings et al., 2009).

## Conclusion and Perspectives

Autism spectrum disorder is a neurodevelopmental disorder and with the increasing incidence of ASD, it is essential to understand what has changed in our genes and environments that may contribute to these disorders. It has been showed that ASD is not a single disease, but rather several conditions including genetic, maternal, and environmental effects that ultimately cause similar behavioral impairments. The abnormalities of ASD may predispose children to various threaten of sleep and make them especially susceptible to sleep problems. Sleep disorders have been reported as one of the most common symptoms and in up to 80% of children with ASD have sleep problems which may even contribute to the altered brain structure and activity (Blakemore et al., 2006). Thus, understanding how sleep affected children with ASD by specific mechanisms such as brain development and synaptic plasticity will enable a broader understanding of the disorders’ causes and provide insights into specific treatments. Over the years, many different sleep analysis methods have been reported. The selection of sleep assessment method should be tailored to specific subjects and taken into consideration of their unique characteristics.

Animal models hold great potential values to investigate the causes and treatments for sleep problems in children with ASD. Numerous animal models of ASD have been generated in the last decade. An ideal animal model should show tight junctions with clinical characteristics of the disease. Rodents are the most common experimental animals and growing studies of NHP models have been generated because their close phylogenetic relatedness to humans.

Because of the complexity and heterogeneity of the ASD, it is still inadequate to understand how genes control and influence complex behavior. The animal models of ASD are currently oversimplified and have many issues. Recently, Liu et al. (2019) demonstrated that an approach to generate cloned monkeys by somatic cell nuclear transfer (SCNT), which can creatively solve the problem of generating NHP models with uniform genetic backgrounds. This study is profoundly improving the overall reproducibility of the model. Continued research used this technique to generate five BMAL1 knockout monkeys for sleeping study, and these monkeys exhibited more activities and reduced sleep during night (Qiu et al., 2019). The development of genome editing technologies (such as CRISPR/Cas9 and base editing, etc.) has opened up the revolutionary ways to directly target and modify genomic sequences in animals (Kang et al., 2019; Zhao J. et al., 2019; Li et al., 2020). We anticipate greater numbers of applications will materialize shortly, such as genome-edited NHP combined with SCNT. Although some issues still need to be solved, studying the sleep disorder across multiple biological scales can offer the hope in the field of translational medicine for ASD and other human diseases. The role of NHP animal model in this process is irreplaceable and must be recognized.

## Author Contributions

All authors listed have made a substantial, direct and intellectual contribution to the work, and approved it for publication.

## Conflict of Interest

The authors declare that the research was conducted in the absence of any commercial or financial relationships that could be construed as a potential conflict of interest.
